# Mobile Apps for Management of Tinnitus: Users’ Survey, Quality Assessment, and Content Analysis

**DOI:** 10.2196/10353

**Published:** 2019-01-23

**Authors:** Magdalena Sereda, Sandra Smith, Kiri Newton, David Stockdale

**Affiliations:** 1 National Institute for Health Research (NIHR) Nottingham Biomedical Research Centre Nottingham United Kingdom; 2 Hearing Sciences, Division of Clinical Neuroscience, School of Medicine, University of Nottingham Nottingham United Kingdom; 3 Division of Audiology, Leicester School of Allied Health Sciences, De Montfort University Leicester United Kingdom; 4 British Tinnitus Association Sheffield United Kingdom

**Keywords:** tinnitus, mobile apps, disease management, surveys and questionnaires, Mobile Apps Rating Scale

## Abstract

**Background:**

Tinnitus is the perception of a sound without any outside source. It affects 6 million people in the United Kingdom. Sound therapy is a core component of many tinnitus management programs. Potential mechanisms of benefit include making tinnitus less noticeable, habituation, distracting attention from tinnitus, relaxation, and promoting neuroplastic changes within the brain. In recent years, there has been a substantial increase in the use of mobile technology. This provided an additional medium through which people with tinnitus can access different tinnitus management options, including sound therapy.

**Objective:**

The aim of this study was to (1) generate the list of apps that people use for management of their tinnitus, (2) explore reasons for app use and nonuse, (3) perform quality assessment of the most cited apps, and (4) perform content analysis to explore and describe options and management techniques available in the most cited apps.

**Methods:**

A Web-based survey consisting of 33 open and closed questions captured (1) demographic information, information about tinnitus, and hearing loss and (2) mobile app–specific information about the motivation to use an app, the apps which respondents used for tinnitus, important factors when choosing an app, devices used to access apps, and reasons for not using apps. The quality of the most cited apps was assessed using the Mobile Apps Rating Scale (MARS). Content and features of the most cited apps were analyzed.

**Results:**

Data from 643 respondents were analyzed. The majority of respondents (482/643, 75.0%) had never used an app for management of tinnitus mainly because of lack of awareness (381/643, 59.3%). The list of the 55 apps that people use for their tinnitus was generated. These included apps that were developed specifically for the management of tinnitus; however, the majority of cited apps were developed for other problems (eg, sleep, depression or anxiety, and relaxation). Quality assessment of the 18 most popular apps using MARS resulted in a range of mean scores from 1.6 to 4.2 (out of 5). In line with the current model of tinnitus management, sound was the main focus of the majority of the apps. Other components included relaxation exercises, elements of cognitive behavioral therapy, information and education, and hypnosis.

**Conclusions:**

People used apps for the management of their tinnitus; however, this was done mostly as a self-help option, without conjunction with management provided by hearing health care professionals. Further research should consider the place for apps in tinnitus management (stand-alone self-management intervention vs part of the management by a hearing professional). As the content of the apps varies with respect to sound options, information, and management strategies, it seems that the choice of the best management app should be guided by individual patient’s needs and preferences.

## Introduction

### Background

Tinnitus is the perception of a sound without any outside source. It affects 6 million people in the United Kingdom. Sound therapy, in the form of hearing aids or sound generators, is a core component of many tinnitus management programs. Potential mechanisms of benefit include making tinnitus less noticeable, promoting habituation, distracting attention from tinnitus, relaxation, and promoting neuroplastic changes within the auditory system. Sound therapy can be provided by a range of media, including hearing aids, wearable sound generators, combination hearing aids, or bedside or tabletop sound generators [1].

Mobile technology, including mobile phone provides an additional medium through which people with tinnitus can access different tinnitus management options, including sound therapy. Recent years have seen a substantial increase in the use of mobile technology. According to the recent Global Mobile Consumer Survey by Deloitte, 85% of adults in the United Kingdom own a mobile phone, and this number is expected to increase to 90% by 2020. More than 37 million people aged 16 to 75 years use their device every day, and 34% look at their device within 5 min of waking [2].

The use of mobile apps to deliver health care (mobile health, mHealth) has several advantages, including (1) improved access to health care, (2) improved quality of health care, and (3) lowering the cost of health care [3]. There are also potential issues associated with mHealth, and these include safety or misuse [4], quality and effectiveness [5,6], responsibility, and risk [7]. The attitudes of patients and health care professionals toward these new developments also need to be assessed and addressed [8].

The quality and functionality of health care apps, including tinnitus apps can vary greatly. The IMS Institute for Healthcare Informatics [9] assessed the functionality of 16,275 health care apps according to 25 individual criteria, including the type and quantity of information provided by the app, how the app tracks or captures user data, the communication processes utilized by the app, and the quantity of device capabilities included in the app. More than 90% of the apps tested received a score of 40 or less out of a possible 100, which indicates the general low quality of the apps tested.

In 2013, the National Health Services (NHS) Commissioning Board created a digital apps library for health care apps. Currently, in the early version of the library, there are 42 apps listed that “meet the high standard of quality, safety, and effectiveness” [10]. Some apps were tested further to assure they meet NHS standards for clinical effectiveness, safety, usability, and accessibility. Although the library includes apps developed for variety of health care conditions as well as healthy living in general, it does not currently list apps for management of tinnitus. However, people with tinnitus might find some of the apps helpful, for example, those developed for the management of stress and anxiety.

To date, no research has looked specifically at the use of mobile apps for tinnitus management. A study by Paglialonga et al [11] identified and assessed apps for hearing science and care in general, which were available on the leading platforms (iOS, Android, and Windows phone stores). Tinnitus apps identified by the authors were mentioned in 2 categories: (1) screening and assessment (estimation of tinnitus pitch and loudness) and (2) intervention and rehabilitation (tinnitus management tools such as maskers and sound stimulation). The identified apps were intended to be used by hearing health care professionals, people with tinnitus, or both.

### Objectives

Despite the increasing popularity of apps in general, it is unclear what proportion of people use apps for tinnitus management and which apps are the most popular. The purpose of this study was to (1) generate the list of apps that people use for management of their tinnitus, (2) explore reasons for apps use and nonuse, (3) perform quality assessment of the most cited apps, and (4) perform content analysis to explore and describe options and management techniques available in these most cited apps.

## Methods

### Web-Based Survey

The Checklist for Reporting Results of Internet e-Surveys was used to report the methods and results of the survey ([12] Multimedia Appendix 1). Ethics approval for the study was granted by the University of Nottingham Faculty of Medicine & Health Sciences Research Ethics Committee reference number LT18082016. As this was an anonymous Web-based survey, completion of the survey was taken as informed consent. No identifiable data were collected.

#### Survey Development

Items for the survey were decided through an iterative process. A list of questions was generated to capture (1) demographic information about respondents (gender, age group, and country of residence); information about tinnitus (presence, duration, and severity); and hearing loss (presence, severity, and use of devices to address hearing loss) and (2) mobile app–specific questions asked about motivation to use an app to manage tinnitus; a list of apps respondents used for managing tinnitus; important factors when choosing an app; devices used to access apps; and reasons for not using apps to manage tinnitus. First, questions were generated in collaboration with the British Tinnitus Association (BTA) and based on the information about the apps that patients were seeking when contacting the BTA. Second, questions were generated to capture information missing from the general tinnitus literature (eg, factors that drive the decision to try apps or factors important when choosing apps for tinnitus management). Questions were first drafted by one of the authors (MS) and then appraised and reduced by other coauthors toward strong face validity and relative merit of the included items. The final questionnaire included 33 items presented on 15 pages. The final survey comprised a mix of open and closed questions and took between 15 and 30 min to complete. The survey used skip logic depending on if a participant had used or not used apps for tinnitus management before or had or did not have tinnitus. No randomization of items was used. All questions, with exception of questions asking about additional comments, were mandatory. Respondents were unable to change their responses once submitted.

#### Administration

Over a 2-month period, people were invited to take part in an anonymous Web-based survey, which was hosted on Survey Monkey (Survey Monkey Inc., San Mateo, California, USA). Responses were collected between August 15, 2016, and November 15, 2016. The survey was open to anyone who wanted to take part, and both app users and nonusers were invited. The survey was advertised via email to current BTA members and National Institute for Health Research (NIHR) Nottingham Biomedical Research Centre (BRC) participants’ database members. The link to the questionnaire was sent out using social media to people following the BTA and BRC via Facebook and Twitter. Only 1 submission from each internet protocol address was permitted by the survey software.

#### Analysis

Closed questions were analyzed in IBM’s SPSS Statistics 24 using descriptive statistics, including frequencies, means, and SDs. Patterns of use depending on age, tinnitus severity and duration, hearing loss, and gender were analyzed using chi-square statistics. Qualitative data from the open questions were analyzed separately using inductive thematic analysis.

### Quality Assessment of the Apps

The quality of the most cited apps listed by respondents was assessed by 3 researchers using the Mobile Apps Rating Scale (MARS) [13]. To be included in the quality assessment, an app needed to be cited by 2 or more people. The MARS scale was developed to be a simple, objective, and reliable tool for assessing the quality of mHealth apps. It contains 23 items rated on a 5-point scale (1=inadequate, 2=poor, 3=acceptable, 4=good, and 5=excellent) or not applicable. A total of 19 questions form the objective quality section, which is divided into 4 scales: engagement, functionality, aesthetics, and information quality. In addition, 4 questions form the subjective quality section evaluating users’ satisfaction. Each app was scored independently by 3 researchers (MS, SS, KN, or DS) using MARS. Apps were tested on Android and iPhone devices where the app was available on both devices. This was followed by a consensus meeting where the scores and reasons for them were discussed. Consensus on the final scoring was then reached by all 3 raters for the objective scales. For the subjective scale, an average rating was taken.

### Content and Features Analysis

Content and features of the most cited apps were analyzed using a bottom-up approach. MS developed a coding manual based on the features listed in the Web-based description of the apps in the Apple App Store, Google Play, and the Amazon App Store, including descriptions and example quotes from the text. The coding manual was reviewed by MS and SS to assure clarity of definitions and examples. A small sample of the cited apps was then assessed by MS, and any missing codes generated were added to the coding manual. This coding manual was then used to identify the content and features of the most cited apps. MS and SS independently applied the coding manual to each mobile app to clarify ambiguous codes, remove duplicate codes, and identify data that did not fit the coding scheme. Coding was then compared and discussed between coders, and subsequent modifications made to the coding manual, resulting in final version of the manual (Multimedia Appendix 2).

## Results

### Participation Data

A total of 675 people responded to the survey. Responses were collected between August 15, 2016, and November 15, 2016. Of the 675 participants who read the welcome page and proceeded to consenting, 671 consented to take part in the survey, which translated to 99.4% (671/675) participation rate. The data were included in the analysis if the respondents provided a response to the question asking if they had ever used an app to manage their tinnitus, which left 643 responses for further analysis. Moreover, 32 people provided only initial demographic information and, therefore, were excluded from the analysis.

Of 643 respondents, 158 respondents had used an app, whereas 485 had never used an app to manage their tinnitus. The majority of participants were UK residents (627/643, 97.5%), with 16 residents from other countries, including Australia (n=5), Canada (n=4), Norway (n=2), Cyprus (n=1), Denmark (n=1), Egypt (n=1), Ireland (n=1), and Malaysia (n=1).

### Demographic Data

**Figure 1:**
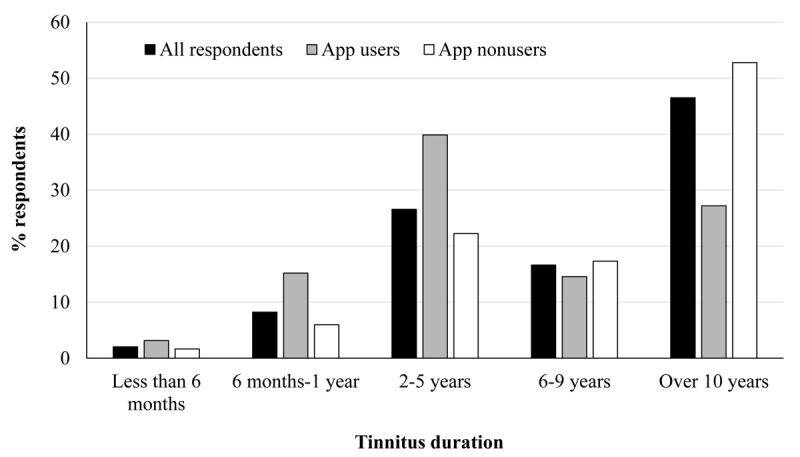
Tinnitus duration in all respondents (black bars), app users (grey bars), and nonusers (white bars).

The majority of respondents (n=637) had tinnitus at the point of completing the survey, whereas 6 had tinnitus in the past. The largest group of respondents were people who had tinnitus for more than 10 years (299/637, 46.9%; Figure 1). There was a significant association between tinnitus duration and use or nonuse of apps, χ^2^_4_=44.8, *P*<.001. Among the users, there were significantly more people in *6 months to 1 year* (*z*=3.0) and *2 to 5 years’* (*z*=3.2) groups and significantly less users in *over 10 years’* group (*z*=−3.6). Among the nonusers, there were significantly more people in the *over 10 years’* group (*z*=2.0).

The majority of respondents (278/673, 41.3%) reported their tinnitus to be moderate, whereas 33 respondents reported slight, 151 mild, and 181 reported severe tinnitus. For the chi-square analysis of tinnitus severity in app users and nonusers, we have combined *slight* and *mild* categories to achieve at least five observations in each category. There was a significant association between tinnitus severity distribution and use or nonuse of apps, χ^2^_2_=11.3; *P*=.004. Among users, there were significantly less people who reported slight or mild tinnitus (*z*=−2.8).

**Figure 2:**
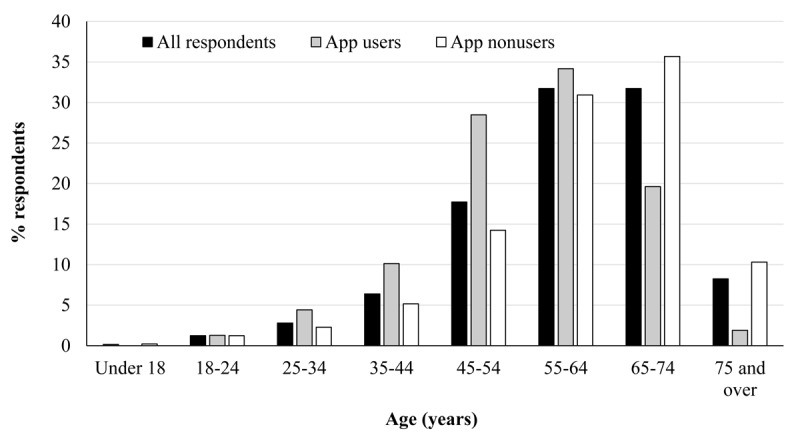
Age distribution for all respondents (black bars), app users (grey bars), and nonusers (white bars).

The age of survey respondents ranged from less than 18 to 75 years and over, with the largest representation from people aged 55 to 64 years (216/675, 32.0%) and 65 to 74 years (216/675, 32.0%; Figure 2). The majority of survey respondents were people with tinnitus; therefore, such age distribution is in line with the data showing higher prevalence of tinnitus with age [14]. There was a significant association between the age distribution and use or nonuse of apps, χ^2^_7_=40.9; *P*<.001. Among the users, there were significantly more people in the 45 to 54 years group (*z*=3.2) and significantly less in the 65 to 74 years (−2.7) and more than 75 years (*z*=−2.8) groups.

Of 643 respondents, 289 (44.9%) were female, 350 (54.4%) were male, and 4 (0.6%) identified in another way. The proportion of males versus females was similar among app users (84/158, 53.1% vs 74/158, 46.9%) and nonusers (267/485, 55.1% vs 218/485, 44.9%; χ^2^_1_=0.43; *P*=.51).

**Figure 3:**
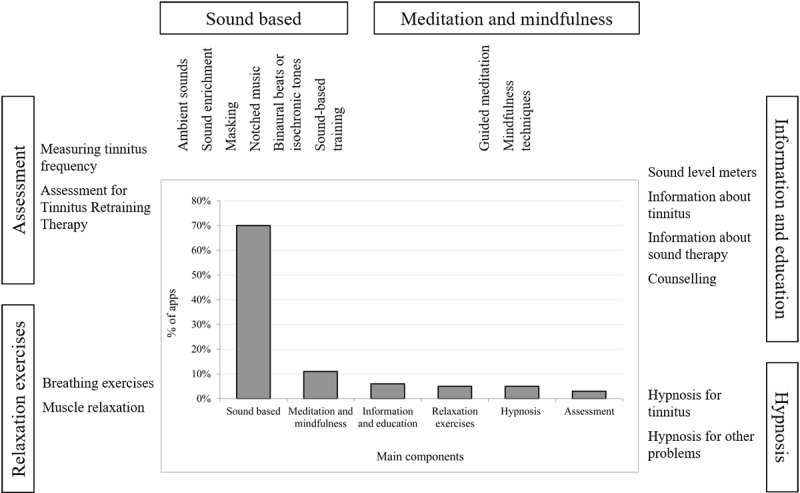
Components of the apps used by respondents for tinnitus management.

The majority of respondents reported some degree of hearing loss (494/643, 76.8%), consistent with the association between tinnitus and hearing loss [15,16]. The largest group of respondents reported mild hearing loss (261/643, 40.6%), with 149 respondents reporting no hearing loss (23.2%), 172 reporting moderate ( /643, 26.7%), and 61 reporting severe or profound hearing loss (61/64, 9.5%). There was a significant association between the degree of hearing loss distribution and use or nonuse of apps, χ^2^_3_=17.5; *P*=.001. There were significantly less app users in the severe or profound hearing loss group (*z*=−2.3).

Of 494 respondents with hearing loss, 263 reported wearing hearing aids and 13 reported wearing cochlear implants. Moreover, 56 hearing aid users and only 2 cochlear implant users reported using apps to manage their tinnitus. In addition, 59 respondents reported using assistive listening devices, and 19 of those reported using apps.

### Apps That People Tried for Tinnitus Management

Altogether, 120 respondents listed 55 apps that they have tried to manage their tinnitus. In addition, 15 people listed apps with a more general context such as radio, YouTube, podcast apps, and audiobook apps, without specifying the exact content that they are using to manage their tinnitus. As there was no way of verifying which contents have been used, those were excluded from further analysis. A full list of the 55 apps listed by respondents and their characteristics is available in Multimedia Appendix 2.

A total of 6 main components of the apps have been identified based on the description in the app stores (Apple, Google, and Amazon): (1) sound generation or therapy, (2) meditation and mindfulness, (3) information and education, (4) hypnosis, (5) relaxation exercises, and (6) assessment (Figure 3). In 70% (38/55) of listed apps, sound was the main focus of the app, including providing a selection of ambient sounds, sound enrichment, sounds for masking distracting sounds or tinnitus, notched music, binaural beats or isochronic tones, and sound-based training. Moreover, 11% (6/55) of apps included guided meditation and mindfulness techniques, and 5% (3/55) of apps had an extensive information and education component and included sound level meters, apps containing information about tinnitus, information about sound therapy, and counseling. Hypnosis for tinnitus or for other problems was a focus of 5% (3/55) of apps. Relaxation exercises such as breathing exercises and muscle relaxation were the components of 5% (3/55) of apps. In addition, 3% (2/55) of apps focused on assessment, including measuring tinnitus frequency and assessment for tinnitus retraining therapy (TRT).

Of 55 listed apps, 14 were developed specifically for tinnitus (Multimedia Appendix 1). In addition, 6 apps used sound or sound therapy to provide relief or distraction from tinnitus (eg, *Tinnitus Therapy Lite, Sound Relief,* and *Tinnitus Therapy Tunes*), 3 apps provided a combination of sound and relaxation exercises (*Beltone Tinnitus Calmer* and *ReSound Relief*), 3 apps implemented specific tinnitus management programs (*TRT* [*iTinnitus*], Progressive Tinnitus Management [*Tinnitus Balance*], and Zen Therapy [*Widex Zen, Tinnitus Management*]), 1 app indicated it was a combination of informational resource and sound therapy (*Starkey Relax*), 1 app used hypnosis (*Overcome Tinnitus*), and 1 app aimed to measure tinnitus pitch (*Tinnitus Measurer*). Moreover, 5 apps were not developed specifically for tinnitus but mentioned tinnitus as one of the possible applications either through masking or without specifying a specific mechanism through which the app might be helpful for tinnitus. A total of 32 apps were developed for sleep, relaxation, concentration, meditation, stress, anxiety, and general well-being and have not mentioned tinnitus as a potential application of the app, and 2 apps were sound level meters.

Each app was listed by between 1 and 21 respondents. The apps that were the most often listed by respondents as the ones they have tried for managing their tinnitus were *White Noise Free* (n=21), *Oticon Tinnitus Sound* (n=13), *Relax Melodies: Sleep Sounds* (n=10), *myNoise* (n=7), and *Tinnitus Therapy Lite* (n=7).

Given that the majority of the apps (n=37) were only mentioned by 1 respondent, we performed a further analysis for those 18 apps that were listed by at least two people (Table 1; for the full list of apps, please see Multimedia Appendix 3). This included quality assessment using MARS [12] and detailed content analysis.

### Reasons for Nonuse

The most commonly listed reason for not using apps for management of tinnitus was lack of awareness of apps (364/485, 75%). In addition, 20.0% (73/364) of respondents declared that they did not use apps as they were not good with technology, 13.2% (48/364) could not find an app that they thought would be helpful for their tinnitus, 11.5% (42/364) said that they did not have mobile phone or tablet, 4.7% (17/364) had only a basic phone that did not support apps or problems with their phone, 3.0% (11/364) did not need to use apps, 2.7% (10/364) did not think the apps would help with their tinnitus, and 2.5% (9/364) used other technologies such as a bedside sound generator or CD player. Other reasons (<1%) for app nonuse included hearing problems, wanting a cure rather than management option, lack of knowledge about which apps could help, not willing to rely on technology, not willing to pay attention to tinnitus, apps exacerbating tinnitus, hyperacusis, preference for a personal contact, too many apps to choose from, having tinnitus for a short period, or lack of interest in apps.

**Table 1:** Characteristics of the apps mentioned by at least three respondents (N=number of times app was cited).

Name	Developer	Category	Star rating^a^	Cost GBP^b^ (£)	In-app purchases	Installs^c^	Platform and version	Last update
White Noise Free (N=21)	TMSOFT	Health and fitness	4.5	Free	No	1-5 million	Apple (7.0), Google (varies with device), and Amazon (7.2.3)	2016
Oticon Tinnitus Sound (N=13)	Oticon A/S	Medical	4.2	Free	No	50,000-100,000	Apple (1.0.2) and Google (1.0.1)	2015
Relax Melodies: Sleep Sounds (N=10)	Ipnos Software	Health and fitness	4.7	Free	Yes	5-10 million	Apple (6.2), Google (varies with device), and Amazon (6.1.2)	2017
myNoise (N=7)	myNoise BVBA	Health and fitness	4.5	Free	Yes	50,000-100,000	Apple (2.4.2) and Google (1.2)	2017
Tinnitus Therapy Lite (N=7)	Sound Oasis	Health and fitness	4.5	Free sample of 5 sounds and basic options	No Pro version available	10,000-50,000	Apple (1.1.6), Google (1.1.6), and Amazon (1.0.3)	2017
Headspace: Guided Meditation & Mindfulness (N=6)	Headspace, Inc	Health and fitness	3.9	Free sample of 10-day meditation	No Pro version available	1-5 million	Apple (3.4.0), Google (3.1.2), and Amazon (2.2.0)	2017
Sleep Bug: White Noise Soundscapes & Music Box (N=6)	Panzertax	Health and fitness	4.4	Free	Yes	100,000-500,000	Apple (3.4) and Google (1.6)	2017
Beltone Tinnitus Calmer (N=4)	Beltone	Medical	4.3	Free	No	10,000-50,000	Apple (3.4.2) and Google (3.1.4)	2017
Sleep Pillow (N=4)	FITNESS22 LTD	Health and fitness	4.8	Free	Yes	100,000-500,000	Apple (7.4) and Google (4.3)	2016
Soothing Sounds Lite (N=4)	Lost Ego Studios Limited	Apple: Medical and Google: Lifestyle	3.5	Free	No	1000-5000	Apple (1.22) and Google (1.0)	2017

^a^Average star rating across platforms.

^b^GBP: Great Britain Pound/Pound Sterling

^*c*^Data on number of installs were available only on Google Play.

### Motivation to Try an App

Of the 158 respondents, 36 (22.8%) of respondents tried an app to address sleep problems, including getting to sleep and staying asleep, 33 (20.9%) hoped to achieve masking of their tinnitus, and 31 (19.6%) followed a recommendation from a hearing professional, family member, or people on the Web. Moreover, 29 (18.4%) tried an app to achieve more general goals such as tinnitus relief, find ways of managing their tinnitus, help with tinnitus, and coping with tinnitus, without specifying ways or mechanisms through which that could be achieved. For 15 (9.5%) respondents, the motivator to try an app was desperation and frustration because of tinnitus, and 14 (8.9%) were looking for a source of sound generation and sound enrichment. In addition, 12 (7.6%) respondents reported that they had tried an app because of the convenience (ie, when they travel because of portability), and 10 (6.3%) looked for an alternative to other technologies such as CDs, radio, pillow speakers, or combination aids. Other motivators for trying an app (<5%) included achieving tinnitus reduction or alleviation, distraction, relaxation, reducing stress or anxiety, having an option to stream via hearing aid, variety or choice of sounds in the app, curiosity, free trial of an app, and aiding habituation.

### Important Factors When Choosing an App

Ease of use (87/120, 72.5%), followed by trustworthy source (53/120, 44.2%), reviews (47/120, 39.2%), and cost (47/120, 39.2%) were most commonly listed as important factors when choosing an app. Additional factors included recommendation by a medical professional (31/120, 25.9%), recommendation by another person with tinnitus (22/120, 18.3%), and recommendation by friend or family (7/120, 5.9%), followed by name of an app (4/120, 4.2%).

### Mobile Apps Rating Scale App Quality Scores

Overall, 3 researchers rated the apps and reliability of objective scales calculated as Cronbach alpha before consensus was .76. Consensus was reached on all the ratings for all the rated apps. Table 2 presents final scores for the 4 subscales (engagement, functionality, aesthetics, and information), overall quality score (mean of 4 subscales), and subjective quality score (satisfaction) for the 18 apps that at least two respondents listed as those that they have tried for management of their tinnitus. Overall, the average MARS quality scores for 18 apps mentioned by at least two respondents varied from 1.5 to 4.2 (out of 5), with scores for individual subscales varying from 1 to 4.6 (Table 2). Subjective scores varied from 1 to 4.1. Of the 4 subscales, functionality had the highest median score (4.4) and aesthetics had the lowest median score (3.15). The *White Noise Free* app had the highest overall MARS score (4.2), followed by *Relax Melodies* (4.1), *Headspace* (4.1), *Oticon Tinnitus Sound* (3.9), and *Sleep Pillow* (3.9). All but 2 apps (*Soothing Sounds Lite* and *Sleep well Hypnosis)* met or exceeded the minimum acceptability score of 3.0.

### Characteristics of the Most Popular Apps

The characteristics of the 18 most often mentioned apps (listed by at least three respondents) are summarized in Table 1. Those included 6 apps developed specifically for tinnitus (*Beltone Tinnitus Calmer, Oticon Tinnitus Sound, ReSound Relief, Tinnitus Aid, Tinnitus Balance,* and *Tinnitus Therapy Lite*), 4 apps that were developed for other problems but mentioned tinnitus as one of the possible apps (*myNoise, Relax Noise 3, Soothing Sounds,* and *White Noise Free*), and 8 apps that were developed for other problems and did not mention tinnitus (*Nature Sounds, Rain Rain Sleep Sounds, Relaxed Melodies, Headspace, Sleep Bug, Sleep Pillow, Soothing Sounds Lite,* and *Sleep Well Hypnosis*). *Beltone Tinnitus Calmer, Oticon Tinnitus Sound, ReSound Relief,* and *Tinnitus Balance* were developed by hearing aid manufacturers and included an information that they should be used as a part of a tinnitus management plan provided by hearing care professional.

The tinnitus-specific goals listed by the apps included masking tinnitus (*myNoise, Relax Noise 3, Tinnitus Therapy Lite,* and *White Noise Free*), decreasing the annoyance of tinnitus (*Oticon Tinnitus Sound*), providing temporary relief from tinnitus (*Oticon Tinnitus Sound*), shifting attention away or distracting from tinnitus (*Beltone Tinnitus Calmer, Oticon Tinnitus Sound,* and *ReSound Relief*), managing tinnitus using sound therapy (*Tinnitus Therapy Lite*), helping prevent problems associated with tinnitus (*Soothing Sounds Lite*), easing the problems associated with tinnitus (*Soothing Sounds*), and relieving tinnitus symptoms (*Tinnitus Aid*).

Apps that were not developed specifically for tinnitus but mainly for other problems usually listed multiple goals. A total of 9 apps (*myNoise, Nature Sounds, Rain Rain Sleep Sounds, Relax Melodies, Sleep Bug, Sleep Pillow, Sleep Well Hypnosis, Soothing Sounds,* and *White Noise Free*) addressed sleep problems, including falling and staying asleep, insomnia, and improving quality of sleep. Moreover, 5 apps listed relaxation (*Rain Rain Sleep Sounds, Relax Melodies, Sleep Well Hypnosis, Soothing Sounds,* and *White Noise Free*), and 5 apps (*myNoise, Relax Melodies, Sleep Well Hypnosis, Soothing Sounds,* and *White Noise Free*) listed reducing stress or anxiety as one of the goals. Overall, 5 apps included the aim to block distractions or background noises or mask interruptions or noises one disliked (*myNoise, Relax Noise 3, Sleep Bug, Soothing Sounds,* and *White Noise Free*). Three apps listed increasing focus or improving concentration (*myNoise, Sleep Bug,* and *White Noise Free*), and 2 apps aimed to enhance or increase privacy (*Sleep Bug* and *White Noise Free*). Other listed aims included pacifying fussy and crying babies (*White Noise Free*); soothing headaches and migraines (*White Noise Free*); living a healthier, happier, more enjoyable life (*Headspace*); and helping calm a busy mind (*Sleep Bug*).

All the 18 apps had a free version available; however, *Headspace* and *Tinnitus Therapy Lite* had only a limited demonstration of meditations or sounds available for free, with an option to purchase the pro version. The *Tinnitus Aid app* was a free version of the *Tinnitus HQ app*, with more sounds to choose from and more filters available in the pro version. Of the 18 apps, 7 apps had in-app purchases, which allowed the purchase of a larger selection of sounds and of unlocking more advanced options.

**Table 2:** The Mobile App Rating Scale mean scores for 18 most cited apps.

Apps	Engagement	Functionality	Aesthetics	Information	Mean	Subjective
White Noise Free	4.2	4.5	4.3	3.7	4.2	4.1
Oticon Tinnitus Sound	3.6	4.5	4	3.4	3.9	3.1
Relax Melodies	4.4	4.5	3.3	4.2	4.1	4
myNoise	3.8	3.25	3	3	3.3	2.6
Tinnitus Therapy Lite	2.2	5	3.3	2.6	3.3	2.6
Headspace	4.6	4.5	3.3	4.2	4.1	4.1
Sleep Bug	3.4	4	3.7	3.3	3.6	2.6
Beltone Tinnitus Calmer	4.6	3.8	3	3.6	3.8	3.3
Sleep Pillow	3.2	5	4	3.3	3.9	3
Soothing Sounds Lite	1.6	2.3	1.7	1	1.6	1
Tinnitus HQ	3.6	3.5	2.7	3.3	3.3	2.4
Tinnitus Balance	3.2	4	2.7	3	3.2	2.3
Rain Rain Sleep Sounds	2.6	4.3	3	3	3.2	2.25
Nature Sounds	3.2	5	3.3	2.7	3.5	2.5
Relax Noise 3	2.4	5	2.7	3.4	3.4	2
ReSound Relief	4.6	3.8	3	3.6	3.8	3.3
Sleep Well Hypnosis	2.6	4	2.3	2.6	2.9	1.9
Zenways	2.6	5	3.3	2.5	3.4	2.4

Overall, 16 apps were updated in 2016 or 2017, with *Zenways* last updated in 2013 and *Relax Noise 3* in 2015. The number of installs ranged from 1000 to 5000 (*Soothing Sounds Lite*) to 5 to 10 million (*Relax Melodies*). The majority of the apps were classified in the health and fitness category (n=11), with 5 apps (*Beltone Tinnitus Calmer, Oticon Tinnitus Sound, ReSound Relief, Tinnitus Aid,* and *Tinnitus Balance*) in the medical category, 1 app (*Zenways*) in Lifestyle, and 1 app classified as medical in the App store and as Lifestyle in Google store. *Oticon Tinnitus Sound* was the only app stating an age limit, which was more than 17 years.

### Content and Features of the Apps

Detailed content analysis was conducted for those 18 apps that at least two respondents listed as those they have tried to manage their tinnitus. Full list of apps content and features is available in Multimedia Appendix 4.

#### Sound

All but 2 apps (n=16) featured sound generation or sound therapy. Sound generation features included a wide selection of sounds. Overall, 1 app offered the possibility to record and loop your own sounds (*White Noise Free*), 7 apps offered an option to import or download additional sounds for free (*Beltone Tinnitus Calmer, myNoise, ReSound Relief,* and *White Noise Free*) or purchase sounds (*Sleep Pillow*) from the app library or your own library (*Oticon Tinnitus Sound* and *Tinnitus Balance*). The *White Noise Free* app linked to the *White Noise Market* app for even more choice of sounds to download. All 16 apps featured volume control for the sounds. Of the 16 apps, 6 apps allowed adjustments beyond volume control such as adjusting sound balance (*Beltone Tinnitus Calmer, ReSound Relief,* and *White Noise Free*), pitch (*White Noise Free*), frequency shaping (*my Noise* and *Tinnitus Aid*), variance (*White Noise Free*), speed (*White Noise Free*), and intensity (eg, a small log fire to a roaring beach fire and *Soothing Sounds Lite*). *myNoise* had a range of frequency shaping options such as animating sounds (ie, zen, subtle, moderate, allegro, and wobbler), setting color of the sound (brown, grey, pink, and white), setting frequency bandwidth (eg, centered around specific frequency), and scene (eg, dark rain, fairy rain, and under the leaves for Rain Noise). Moreover, 15 apps offered endless sounds, with 3 apps using an option to loop sounds (*Relax Melodies, Tinnitus Balance,* and *White Noise Free*) and *Soothing Sounds*, claiming they were using an advanced soundscape generator, which does not loop sounds but generates them in a way that one would not hear the same 10 seconds of sound. In addition, *Relax Melodies* featured a loop correction option, which allowed the user to try other modes in case the pause could be heard in the looped sounds. The remaining 12 apps have not specified how they have achieved the endless sound. *Tinnitus Aid* offered *long high-quality recordings*.

Overall, 9 apps included the possibility to mix different sounds to create personalized *soundscapes*, with all those apps allowing to adjust the volume of the sounds individually and some of the apps allowing to adjust balance (*Beltone Tinnitus Calmer, ReSound Relief,* and *White Noise Free*) and pitch of individual sounds in the mix (*White Noise Free*). In total, 2 apps allowed users to add random sound effects to the sounds (*Nature Sounds* and *Sleep Bug*). *myNoise* had an option to mix sound available only on iPhone.

Overall, 5 apps allowed to rate or mark the favorite sounds and store them in the favorite folder. Of the 5 apps, 4 apps (*Beltone Tinnitus Calmer, Oticon Tinnitus Sound, ReSound Relief,* and *Tinnitus Balance*) allowed the user to create a personalized sound plan and organize the sounds according to sound type, for example, soothing, interesting, or background (*Beltone Tinnitus Calmer, Oticon Tinnitus Sound, Tinnitus Balance,* and *ReSound Relief*) or according to situations when a particular sounds are preferred (*Oticon Tinnitus Sound* and *Tinnitus Balance*).

In total, 4 apps included binaural beats or isochronic tones in the free versions (*myNoise, Relax Melodies, Soothing Sounds,* and *Zenways*). *Relax Melodies* offered 6 different frequencies, between 2.5 and 20 Hz, of binaural beats, which can affect the brain in different ways. For example, the description in the app suggests that 2.5-Hz delta wave *helps you reach the deepest portion of your sleep cycle*, whereas 10-Hz mid-alpha wave *helps to calm and relax your mind after you have been active*. *myNoise* includes *Binaural Beat Machine*, with 10 carriers between 1 and 32 Hz to induce a particular mental state (eg, deep sleep, relaxed, conscious, and focused). *Soothing Sounds Lite* contained binaural sounds to *help improve concentration as well as relaxation*, with a slider to adjust tones’ frequency.

Overall, 12 apps had a feature to play sound in the background while using other apps (*Beltone Tinnitus Calmer, myNoise, Oticon Tinnitus Sound, Rain Rain Sleep Sounds, Relax Melodies, Relax Noise 3, ReSound Relief, Sleep Bug, Sleep Pillow, Tinnitus Aid, Tinnitus Balance,* and *Zenways*).

*Tinnitus Balance* app used sound in the context of specific management program (Progressive Tinnitus Management).

#### Meditation and Mindfulness

A total of 5 apps featured meditation and mindfulness (*Beltone Tinnitus Calmer, Headspace, Relax Melodies, ReSound Relief,* and *Zenways*), with all 5 featuring guided meditation and 2 using imagery (*Beltone Tinnitus Calmer* and *ReSound Relief*). In 2 apps, meditation and mindfulness was the main focus of the app (*Headspace* and *Zenways*), whereas, in 3 apps, it was one of the features alongside other components.

*Relax Melodies* offered guided meditation programs and single sessions to help sleep. *Beltone Tinnitus Calmer* and *ReSound Relief* offered 6 guided meditation sessions to practice techniques for managing stress and tension caused by tinnitus. *Headspace* offered a wide selection of themed meditations on a variety of topics (eg, depression, self-esteem, stress, cancer, sleep, pregnancy, and anxiety); however, it is worth noting that the only free option is a 10-day meditation program that *taught you the essentials of living a healthier, happier life*. *Zenways* offers mindfulness of the breath meditation to *help relax and find your Zen*.

#### Relaxation Exercises

A total of 3 apps included relaxation exercises (*Beltone Tinnitus Calmer*, *Oticon Tinnitus Sound*, and *ReSound Relief*) alongside other components. All 3 apps had breathing exercises, where the task was to breathe in sync with expanding and collapsing bauble in the screen, together with the voice asking you to breathe in and breathe out. There was also an option to set the tempo of the breathing as deep, slow, or normal in *Beltone Tinnitus Calmer* and *ReSound Relief* and number of breaths per minute in *Oticon Tinnitus Sound*. *Oticon Tinnitus Sound* also featured muscle relaxation, which asks to tense and relax certain group of muscles according to spoken instructions. As all 3 apps containing relaxation exercises were apps developed specifically for tinnitus, the aim of the relaxation was to counteract tension and stress caused by tinnitus and in return notice tinnitus less.

#### Elements of Cognitive Behavior Therapy

Two apps included elements of cognitive behavioral therapy (CBT; *Beltone Tinnitus Calmer* and *ReSound Relief*). One of those apps was changing unpleasant thoughts about tinnitus into something less upsetting, including lack of help, tinnitus ruining life, loud tinnitus or bad day, lack of support from partner, tinnitus getting worse, and lack of understanding. The second CBT element was pleasant activities, where the users are asked to nominate activities they would like to do such as meet a friend for tea, learn a new skill, or play music and receive weekly reminders to do them, on the basis that doing things they enjoy makes life with tinnitus easier.

#### Information and Education

Information and education within the apps included information about tinnitus, using sound for the management of tinnitus, including binaural beats, sleep hygiene, insomnia and its causes, and meditation and mindfulness. *Beltone Tinnitus Calmer* and *ReSound Relief* apps provided information about tinnitus. The apps included the separate section entitled “What is tinnitus?,” covering such topics as how tinnitus is defined, prevalence of tinnitus, causes of tinnitus, what can be done, how to live with tinnitus, and common therapies. *Tinnitus Balance* app contains brief information regarding the prevalence of tinnitus. Overall, 5 apps contained information about using sound for management of tinnitus. *Beltone Tinnitus Calmer, Oticon Tinnitus Sound, ReSound Relief*, and *Tinnitus Balance* explain the different role that soothing, interesting, and background sounds can play in the management of tinnitus. *Tinnitus Therapy Lite* contained a description of their tinnitus relief sounds. *Beltone Tinnitus Calmer* and *ReSound Relief* offered information about sleep hygiene, including sections on eating and drinking, relaxing before bedtime, sleep behavior, sleeping environment, and timing. *Sleep Well Hypnosis* included a spoken introduction at the beginning of the hypnosis session, explaining what is insomnia and possible causes of insomnia. *Relax Melodies* app described the role of different frequencies of binaural beats from 2.5 to 20 Hz. *myNoise* included an explanation of what binaural beats do, but this was only available on the iPhone. The *Headspace* app had a link to a short video explaining what mediation and mind training is.

Overall, 7 apps had weblinks to more information or app help and troubleshooting. *Tinnitus Therapy Lite* included a link to the Sound Oasis website, including more information on tinnitus and how sound therapy can help. *Beltone Tinnitus Calmer* and *ReSound Relief* included a link to the ReSound GN website, with information about their hearing and tinnitus products, links to national tinnitus charities and associations, treatment centers, and information resources about tinnitus management options. *Sleep Well Hypnosis* included a link to answers to *hypnosis questions* such as “how long will it take to notice changes,” “how does hypnosis work,” and “will I lose control while I am under hypnosis.” *myNoise* had a link to *myNoise* on the Web, with detailed information about noise generators, their calibration, extensive sounds library, and using noise for different purposes (at the office, studying, tinnitus, hyperacusis, and relaxation). *Relax Noise 3* had a link to the *Relaxed Noise 3* website, with information about the 3 different types of noises used in the app: white, pink, and red, using those sounds to aid concentration, as tinnitus maskers or noisers, for meditation and as a sleeping aid. *Sleep Well Hypnosis* app features Sleep Booster with binaural beats *to induce your brainwave frequency into an optimal state for deep, restorative sleep*; however, that option is only available in the pro version of the app. Moreover, 6 apps included a help section or brief introduction to an app within the app (available offline).

#### Hypnosis

*Sleep Well Hypnosis* features a single 25-min hypnosis audio session read by a certified hypnotherapist, which aims to *help reduce anxious thoughts and prepare the mind for deeper, more restorative rest*. This can be combined with background music and sleep booster, with binaural beats (only in pro version).

#### Nonauditory Stimuli

*Beltone Tinnitus Calmer* and *ReSound Relief* contain some secondary stimuli, that is, colors. The role of those was described as *“* keeping your mind occupied.” For each of the soundscapes, there was a possibility of choosing *color mood*, which would be displayed while playing the sound.

*Nature Sounds, Sleep Bug, Sleep Pillow,* and *Tinnitus Aid* pointed in the app descriptions to using high-quality graphics. However, some other apps that did not explicitly specify that feature also featured high-quality images (eg, *White Noise Free*).

#### Technical Features

All 18 apps did not require streaming, but instead the content was downloaded to the device and worked offline. Of the 18 apps, 7 apps (*Beltone Tinnitus Calmer, Oticon Tinnitus Sound, Relax Melodies, ReSound Relief, White Noise Free, Sleep Bug,* and *Sleep Well Hypnosis*) had remote controls allowing to adjust volume (*White Noise Free*) and/or pause or start or close the apps while on the screen lock.

Moreover, 5 apps had different options for sharing, with *White Noise Free* featuring the most advanced sharing options of all the apps. These included the possibility of the user sharing their own recordings and mixes and photos. Sharing recordings or mixes is possible via *White Noise Market app*, which connects you to an app community or via email. *Headspace* allows you to invite up to 5 *buddies* through an email message, sends the information about the app, a short video, and link to the website. The *buddies* system allows you to access your buddies’ statistics and progress and motivate them if they fail to meet the goals. *Relax Melodies, Sleep Well Hypnosis, Tinnitus Aid,* and *Zenways* had an option to share the link to the app, for example, via email or messaging apps. *myNoise* and *White Noise Free* featured their own app communities where you can upload, download, and/or rate different sounds and/or post comments.

Overall, 13 apps were advert free (*Beltone Tinnitus Calmer, Headspace, myNoise, Nature Sounds, Oticon Tinnitus Sound, Rain Rain Sleep Sounds, Relax Noise 3, ReSound Relief, Sleep Bug, Sleep Well Hypnosis, Tinnitus Balance, Tinnitus Therapy Lite,* and *Zenways*), whereas 3 apps featured advertisements on the small stripe at the top or bottom of the screen, not interfering with the apps content (*Relax Melodies, Sleep Pillow,* and *White Noise Free*). *Soothing Sounds Lite* was the only app where the adverts took considerable space on the screen, making it difficult to navigate.

*Beltone Tinnitus Calmer*, *Headspace*, *Relax Melodies*, *ReSound Relief*, and *Tinnitus Balance* offered progress or usage tracking. *Headspace* captured the total time spent on meditation, number of sessions completed, and average duration. *Beltone Tinnitus Calmer* and *ReSound Relief* captured total hours used and separately time spent using sounds and exercises. *Tinnitus Balance* reported average usage per day, percentage time spent on sounds sorted according to sound type and sounds sorted according to situation down, to percentage of time spent listening to individual sounds. *Relax Melodies* had an option to track *mindful minutes* using the Apple Health app.

A total of 10 apps were available in multiple language options in addition to English (*Beltone Tinnitus Calmer, Nature Sounds, Oticon Tinnitus Sound, Rain Rain Sleep Sounds, Relax Melodies, ReSound Relief, Sleep Pillow, Soothing Sounds Lite, Tinnitus Aid,* and *Tinnitus Balance*) and *White Noise free* was available in English and Spanish.

Overall, 15 apps featured a timer for controlling the length of the sound or meditation sessions (*Zenways*). Some of them had an option to fade audio out (*Relax Noise 3, Tinnitus Aid,* and *White Noise Free*). The *Headspace* app did not feature a timer and had a predefined meditation sessions length. *Soothing Sounds Lite* did not have a timer meaning that sounds would play until they were turned off. The timer option was displayed but not accessible in the free version of *Sleep Well Hypnosis* app. Moreover, 3 apps had a clock (*White Noise Free, Sleep Bug,* and *Relax Melodies* *—* iPhone only), 3 had alarms (*White Noise Free, Soothing Sounds Lite,* and *Relax Melodies* *—* iPhone only), and 1 had date display (*Sleep Bug*). Two apps (*Rain Rain Sleep Sounds* and *Relax Melodies*) featured bedtime reminders allowing the user to set days and times for going to sleep.

In the description in the app store, *White Noise Free* claimed to feature swipe gesture support for navigating sound collection, it is not clear, however, how that differed from other tested apps. *Sleep Bug* claimed to use accessibility support but did not specify in what way. *Sleep Bug* claimed *great user support* but again did not clarify what it would feature.

## Discussion

### Principal Findings

This study generated the list of 55 apps that people used for the management of their tinnitus and explored reasons for app nonuse as well as motivators for using apps for tinnitus management. The main reason for app nonuse was lack of awareness of their existence. Quality assessment of the 18 most popular apps using MARS resulted in a range of mean scores from 1.6 to 4.2 (out of 5), depending on an app. Sound was the main component of the majority of the apps chosen by people with tinnitus.

The data from the Office of National Statistics [17] showed an increasing use of the internet by those aged over 65 years despite that this group has been consistently the lowest users of internet over the years. A similar pattern was found for apps’ use, with the app users group being slightly younger than the apps nonusers group. The users group showed a lower proportion of people aged 65 years and older and a higher proportion of people aged 45 to 54 years. However, there was still a considerable proportion of people aged over 65 years who were app users.

People listed a large number of apps used for tinnitus management. A majority of the 55 apps were mentioned by only 1 person. There might be several reasons for such variability. First, people were looking for apps for a range of different reasons, including helping with sleep, masking, sound enrichment, distraction, relaxation, and reducing stress or anxiety; therefore, they were choosing apps that were addressing those specific goals or problems. This also explains why the majority of apps used by people were developed for other problems rather than for tinnitus. Second, only 19% of respondents reported that they followed a recommendation when choosing an app, suggesting that the majority of respondents found apps through a search in the app stores. A quick search for tinnitus apps in the Google Play Store returned 248 apps available for download. The large number of apps that can potentially be useful for the management of tinnitus, although encouraging, also poses a challenge for people with tinnitus and hearing care professionals equally. The search results for apps in the app store can be overwhelming, with several hundred apps available when searching for *tinnitus*. Without a clear criteria or guidance on which apps to choose, it is not surprising that people tended to choose different apps based on the reviews or personal preference. Given that ease of use was listed as the most important factor when choosing an app, it would not be surprising if personal preference played a main role in the choice of apps.

Average MARS quality scores for the 18 most cited apps varied greatly with 2 apps not meeting the minimum acceptability score of 3. None of the apps received the maximum score of 5. The lowest median score was for the aesthetics subscale, which asked questions about layout, graphics quality, and visual appeal. The Functionality subscale had a highest median score, with questions about performance, ease of use, navigation, and gestural design. This is in line with the results of previous studies using MARS for quality assessment of weight management apps [18], prevention of driving after drinking alcohol apps [19], and mindfulness-based apps [20], where functionality scores were also the highest.

In line with the current model of tinnitus management, where sound is the main component of the majority of tinnitus management strategies and programs, sound was also the main focus of the majority of apps. Current sound therapy options available on the NHS include various devices that play sounds, including sound generators and combination aids. However, those devices can only play a limited number of sounds; therefore, not all patients find an option that helps with their tinnitus. As the choice of sound options that can be delivered via many of the apps is large and very often sounds are customizable, it is much more likely that the individual patient’s needs regarding sound therapy will be met via this option, allowing for personalization of a tinnitus management plan.

About 20% of people with tinnitus experience symptoms that affect their quality of life. They might experience disturbed sleep, hearing and concentration problems, social isolation, anxiety, depression, irritation, or stress. It is, therefore, not surprising that people listed apps addressing those problems through meditation and mindfulness, relaxation exercises, and elements of CBT. However, it was noted during the quality assessment using the MARS scale that some of the content might not be appropriate for people with tinnitus to access without guidance from a health care professional. Specifically, *Beltone Tinnitus Calmer* and *ReSound Relief* apps have a section giving examples of negative thoughts, which without a proper explanation might potentially have a negative impact on the user.

### Strengths and Limitations

This study is the first one to review mobile phone apps for the management of tinnitus. It is the first study to assess the quality of apps used for tinnitus management using the MARS scale. Apps were tested on both iPhone iOS and Android platforms. Expert ratings on 30% of the reviewed apps had a high-level interrater reliability in this study.

Given the large number of apps for tinnitus management and the fact that people with tinnitus use both tinnitus-specific apps and apps developed for other problems, we have undertaken a bottom-up approach, rather than a systematic search in the apps stores. The strength of such approach is that we were able to identify apps not developed specifically for tinnitus that people use and that might potentially be useful for tinnitus management. Moreover, one of these apps that would not be identified by simple search was *Headspace*: Guided Meditation & Mindfulness. On the other hand, there might be some apps that were missed from our list. Given that this is the first study looking at apps for tinnitus, it seemed the best approach to, in the first instance, look at the apps currently used by people with tinnitus.

### Future Research

Our study showed that people use apps for the management of their tinnitus; however, this is done mostly as a self-help option, without conjunction with management provided by hearing health care professionals. Future research should look at the possibility of incorporating apps into the management of tinnitus by health care professionals and creating guidelines for the use of apps as a part of a tinnitus management plan. Further research involving patients and clinicians on the desired content and usability features of apps for tinnitus management should be conducted. There is no evidence for the efficacy of apps for the management of tinnitus, and none of the listed apps were assessed for efficacy for tinnitus management in a trial. Future research is needed to determine the efficacy of apps for management of tinnitus.

### Conclusions

Further research should consider the place for apps in the tinnitus management (stand-alone self-management intervention vs part of the management by a hearing professional). As content of the apps varies in respect to sound options, information, and management strategies, it seems that the choice of the best management app should be guided by individual patient needs and preferences.

## Appendix

Multimedia Appendix 1 Checklist for Reporting Results of Internet E-Surveys (CHERRIES).

Multimedia Appendix 2 Coding manual developed for content analysis of the apps.

Multimedia Appendix 3 Characteristics of the 55 apps listed by respondents as those they have used for management of their tinnitus.

Multimedia Appendix 4 Content analysis and features of the 18 apps that at least two respondents listed as those they have tried to manage their tinnitus: (1) White Noise Free, (2) Oticon Tinnitus Sound, (3) Relax Melodies: Sleep Sounds, (4) myNoise, (5) Tinnitus Therapy Lite, (6) Headspace: Guided Meditation & Mindfulness, (7) Sleep Bug: White Noise Soundscapes & Music Box, (8) Beltone Tinnitus Calmer, (9) Sleep Pillow, (10) Soothing Sounds Lite, (11) Tinnitus Aid: Nature sounds to mask ear ringing, (12) Tinnitus Balance, (13) Rain Rain Sleep Sounds, (14) Nature Sounds, (15) Relax Noise 3, (16) ReSound Relief, (17) Sleep Well Hypnosis, and (18) Zenways. V—content or feature present in an app.

## Abbreviations

BRC: Biomedical Research Center
BTA: British Tinnitus Association
CBT: cognitive behavioral therapy
MARS: Mobile Apps Rating Scale
mHealth: mobile health
NHS: National Health Service
NIHR: National Institute for Health Research
TRT: tinnitus retraining therapy
