# Supplementary material for: Mobile Apps for Management of Tinnitus: Users’ Survey, Quality Assessment, and Content Analysis
Source: JMIR Mhealth Uhealth. 2019 Jan 23;7(1):e10353. doi: 10.2196/10353 (PMC6364200; doi:10.2196/10353)
Supplement: Multimedia Appendix 1 [file mhealth_v7i1e10353_app1.pdf]

Multimedia Appendix 1. Checklist for Reporting Results of Internet E-Surveys (CHERRIES)

**Item category**	**Checklist item**	**Page no.**	**Description**
Design	Study design	5	Over a 2-month period, people were invited to take part in an anonymous online survey which was hosted on Survey Monkey©. The survey was open to anyone who would like to take part and both app users and non-users were invited.
Ethics	Ethics approval	4	Ethics approval for the study was granted by the University of Nottingham Faculty of Medicine & Health Sciences Research Ethics Committee ref no. LT18082016.
	Informed consent	4	As this was an anonymous online survey completion of the survey was taken as informed consent.
	Data protection	4	No identifiable data were collected.
Development and Pre-testing		4/5	Items for the survey were decided through an iterative process. First, questions were generated in collaboration with British Tinnitus Association (BTA) and based on information about the apps that patients are seeking when contacting the BTA. Second, questions were generated to capture information missing from the general tinnitus literature (e.g. factors that drive the decision to try apps or factors important when choosing apps for tinnitus management). Questions were first drafted by one of the authors (MS) and then appraised and reduced by other co-authors towards strong face validity and relative merit of the included items. The final questionnaire included 33 items. The final survey comprised a mix of open and closed questions and took between 15 and 30 minutes to complete.
Recruitment process	Open vs closed suvey	5	The survey was open to anyone who would like to take part and both app users and non-users were invited.
	Contact mode	5	Survey was advertised via email to current British Tinnitus Association (BTA) members and NIHR Nottingham Biomedical Research Centre (BRC) participants' database members.
	Advertising the survey	5	The link to the questionnaire was sent out using social media to people following the BTA and BRC via Facebook and Twitter.

Survey administration	Web/email	5	The survey was an online survey which was hosted on Survey Monkey©.
	Context	5	The survey was an online survey which was hosted on Survey Monkey©. Survey was advertised via email to current British Tinnitus Association (BTA) members and NIHR Nottingham Biomedical Research Centre (BRC) participants' database members.
	Mandatory/voluntary	5	The survey was open to anyone who would like to take part and both app users and non-users were invited.
	Incentives		Not applicable
	Time/date	6	Responses were collected between 15 August 2016 and 15 November 2016.
	Item randomisation	5	No randomisation of items was used.
	Adaptive Questioning	5	The survey used skip logic depending if participant used/not used apps for tinnitus management before or had/did not have tinnitus.
	Number of items	5	The final questionnaire included 33 items
	Number of screens	5	The final questionnaire included 33 items presented on 15 pages. The final survey comprised a mix of open and closed questions and took between 15 and 30 minutes to complete.
	Completeness check	5/6	All question, with exception of questions asking about additional comments, were mandatory. Six hundred and seventy five people responded to the survey. Responses were collected between 15 August 2016 and 15 November 2016. The data were included in the analysis if the respondents provided response to the question if they ever used an app to manage their tinnitus, which left 643 responses for further analysis. Thirty two people provided only initial demographic information, therefore were excluded from the analysis.
	Review step	5	Respondents were unable to change their responses once submitted.
Response Rates	Unique site visitor	5	Only one submission from each IP address was permitted by the survey software.
	View rate		Not collected
	Participation rate	6	From 675 participants who read the welcome page and proceeded to consenting, 671 consented to take part in

			the survey, which gives 99.4% participation rate.
	Completion rate	6	The data were included in the analysis if the respondents provided response to the question if they ever used an app to manage their tinnitus, which left 643 responses for further analysis. Thirty two people provided only initial demographic information, therefore were excluded from the analysis.
	Cookies used		No
	IP Check	5	Only one submission from each IP address was permitted by the survey software.
	Log file analysis		No
	Registration		Not applicable
Analysis	Handling of incomplete questionnaires	6	The data were included in the analysis if the respondents provided response to the question if they ever used an app to manage their tinnitus, which left 643 responses for further analysis. Thirty two people provided only initial demographic information, therefore were excluded from the analysis.
	Questionnaires with atypical timestamp		Not applicable
	Statistical correction		Not applicable
