# Supplementary material for: Mobile Apps for Management of Tinnitus: Users’ Survey, Quality Assessment, and Content Analysis
Source: JMIR Mhealth Uhealth. 2019 Jan 23;7(1):e10353. doi: 10.2196/10353 (PMC6364200; doi:10.2196/10353)
Supplement: Multimedia Appendix 2 [file mhealth_v7i1e10353_app2.pdf]

Multimedia Appendix 2: Coding manual developed for content analysis of the apps

**Code**	**Example**
Sounds (sound generation)	*White Noise Free* (Google Play, App store, Amazon): Features ambient sounds of environment. 40 sounds are included as part of the application including all the colour noises (brown, white pink etc.), mechanical sounds like bedroom fan or air conditioner, light to heavy rains, rushing water sounds, beach and ocean waves and so much more.
Mixing sounds	*White Noise Free* (Google Play, App store, Amazon): Create new soundscapes by multiple noises together like a DJ with support for adjusting volume, balance, and pitch of individual sounds in the mix.
Loop sounds/sessions	*White Noise Free* (Google Play, App store, Amazon):
Import/download sounds	*White Noise Free* (Google Play, App store, Amazon): Import new sounds for FREE from the White Noise Market at http://white noisemarket.com/
Alarms	*White Noise Free* (Google Play, App store, Amazon): Create multiple alarms that slowly fade in waking you up feeling refreshed.
Timer	*White Noise Free* (Google Play, App store, Amazon): Sound timers turn off audio to save battery life and fade audio out so you don't suddenly awake.
Clock	*White Noise Free* (Google Play, App store, Amazon): Full screen digital clock with multiple colours and brightness controls makes it the perfect companion for any nightstand.
Favourite sounds/mixes	*White Noise Free* (Google Play, App store, Amazon): Star favourite sounds and mixes in the sound catalogue for quick access using the Favourite view.
Gesture support	*White Noise Free* (Google Play, App store, Amazon): Swipe gesture support for navigating sound collection.
Sound volume adjustments	*White Noise Free* (Google Play, App store, Amazon): Adjust sound volume.
Sound adjustments (beyond volume adjustments)	*White Noise Free* (Google Play, App store, Amazon): Adjust sound balance, pitch.<br>*myNoise* (Google Play, App store): Through a simple but accurate calibration process, our calibrated noise generators are not only matched to your own hearing, but also compensate for your audio equipment and listening environment deficiencies, including the presence of background noise and its nature.
No streaming/ works offline	*White Noise Free* (Google Play, App store, Amazon): No streaming is required for playback.<br>*Headspace* (Google Play, App store, Amazon): Future meditations available for download to use anytime, anywhere.
Upload and share	*White Noise Free* (Google Play, App store, Amazon): Upload and share your recordings and mixes with the White Noise Market app.
Wireless options	*White Noise Free* (Google Play, App store, Amazon): AirPlay sounds to Bluetooth devices or Apple TV
Remote controls	*White Noise Free* (Google Play, App store, Amazon): Remote media controls on lock screen and headphones
Sound plan	*Oticon Tinnitus Sound* (Google Play, App store): This app allows you to create a Sound plan. Sounds can be organised according to sound type (soothing, interesting or background sound) or according to situations when a particular sound or sounds are preferred.
Relaxation exercises	*Oticon Tinnitus Sound* (Google Play, App store): The app includes two exercises to help you relax and stay calm. *From the app: Breathing exercise and muscle relaxation.*

Connect to hearing aids - wireless	*Oticon Tinnitus Sound* (Google Play, App store): The app is intended to be used with Oticon wireless hearing tinnitus aids connected to Oticon Streamer Pro.
Meditation and Mindfulness	*Headspace* (Google Play, App store, Amazon): We'll help you perform your best through the skills of meditation and mindfulness.
Progress/usage tracking	Headspace (Google Play, App store, Amazon): reports of your progress throughout the programmes.
Sound effects/Random sound effects	*Sleep Bug* (Google Play, App store): 83 different sound effects. (…) produces a variety of scenes and music with random sound effects.
Play sound in the background	*Sleep Bug* (Google Play, App store): Play scenes in the background while using other apps.
Accessibility support	*Sleep Bug* (Google Play, App store): Accessibility support.
High quality graphics (images)	*Sleep Bug* (Google Play, App store): High quality retina graphics.
No adds	*Sleep Bug* (Google Play, App store): No adds
User support	*Sleep Bug* (Google Play, App store): Great user support.
Date display	*Sleep Bug* (Google Play, App store): Clock and date display.
Binaural beats / Isochronic tones	*Relax Melodies* (Google Play, App store, Amazon): including 6 binaural beat frequencies for brainwave entertainment.
App community	*Relax Melodies* (Google Play, App store, Amazon): 'Community melodies' driven by the best mixes of the community.
Colours	*Beltone Tinnitus Calmer* (Google Play, App Store): Contains some secondary stimuli like colours to keep your mind occupied.
Exercises	*Beltone Tinnitus Calmer* (Google Play, App Store): Contains some secondary stimuli like exercises to keep your mind occupied.
Hypnosis	*Sleep Well Hypnosis* (Google Play, App store, Amazon): Train your mind to sleep better through hypnosis. Hypnosis audio carefully read by the soothing voice of certified hypnotherapist.
Bedtime reminder	Rain Rain Sleep Sounds (Google Play, App store, Amazon): Achieve your bedtime goals with Rain Rain's integrated Bedtime Reminder by setting what time and days you would like to go to sleep. Rain Rain sends you a gentle, encouraging reminder when it is time to go to bed so that you can get the rest you deserve.
Alarm	*Soothing Sounds* (Google Play, App store): Contains (…) alarm.
Not looping, endless sound or new technology	*Soothing Sounds* (Google Play, App store): This app has been designed to be the most advanced soundscape generator available on the Google Play Store. It has fully customisable sound library so you'll never hear the same 10 seconds of sound.
Information about using sound for management of tinnitus	Added after assessment of sample of apps. *Oticon Tinnitus Sound* (Google Play, App store): Sound types and situations. Three Sound types can be useful to manage your tinnitus (…)
Loop correction mode	Added after assessment of sample of apps. *Relax Melodies* (Google Play, App store, Amazon): Try other modes if you hear a pause in sound loops.
Information about binaural beats and isochronic tones	Added after assessment of sample of apps. *Relax Melodies* (Google Play, App store, Amazon): Isochronic tones influence the brain in various ways. The effect of each brainwave depends on its frequency (…)
Elements of CBT	Added after assessment of sample of apps. *Beltone Tinnitus Calmer* (Google Play, App Store): Changing your thoughts can change how you feel. Below are some common upsetting thoughts about tinnitus. Click on a though for suggestions on how to change it into something less upsetting.

Information about sleep hygiene	Added after assessment of sample of apps. *Beltone Tinnitus Calmer* (Google Play, App Store): Below are some tips for getting better sleep. Practicing these tips daily may help you at night.
Information about tinnitus	Added after assessment of sample of apps. *Beltone Tinnitus Calmer* (Google Play, App Store): What is tinnitus?
Tutorial/help/introduction to the app	Added after assessment of sample of apps. *Oticon Tinnitus Sound* (Google Play, App store): The app and you<br>*Beltone Tinnitus Calmer* (Google Play, App Store): Introduction to the app: Sound therapy, Distraction, Usage, Get started.
Sharing	Added after assessment of sample of apps. Relax Melodies (Google Play, App store, Amazon): More – Share (allows sharing link to an app via number of social media).
Record Sounds	Added after assessment of sample of apps. *White Noise Free* (Google Play, App store, Amazon): Mix & Record. Record, loop, and share your own sounds too!
Web link to more information/help	Added after assessment of sample of apps. Beltone Tinnitus Calmer (Google Play, App Store): Additional references (links to: American Tinnitus Association, British Tinnitus Association, New Zealand Tinnitus Association, United States National Library of Medicine, Tinnitus Retraining Therapy etc.).
Clock	Added after assessment of sample of apps. *White Noise Free* (Google Play, App store, Amazon): Sleep clock. It’s the perfect companion to your nightstand.
