# Supplementary material for: Mobile Apps for Management of Tinnitus: Users’ Survey, Quality Assessment, and Content Analysis
Source: JMIR Mhealth Uhealth. 2019 Jan 23;7(1):e10353. doi: 10.2196/10353 (PMC6364200; doi:10.2196/10353)
Supplement: Multimedia Appendix 3 [file mhealth_v7i1e10353_app3.pdf]

Multimedia Appendix 3: Characteristics of the 55 apps listed by respondents as those they have used for management of their tinnitus

Name	Developer	Version	Category	Star rating*	Cost GBR £	In app purchases	Installs	Last update	No of times cited	Tinnitus specific	Main Focus
White Noise Free	TMSOFT	7.3. Amazon<br>7.2. Apple<br>7.0 google	Health & Fitness	4.5	Free	No	1-5 million	2016	21	**Mentions tinnitus**: features all of kinds of quality noise samples **helping mask your tinnitus**	Sleep, reduce stress, increase privacy, and mask your tinnitus, concentration, blocking distractions.
Oticon Tinnitus Sound	Oticon A/S	1.0.2 Apple<br>1.0.1 google	Medical	4.2	Free	No	50-100k	2015	13	**Tinnitus specific:** selection of sounds to **decrease the annoyance of tinnitus**, to provide temporary relief	Tinnitus
Relax Melodies: Sleep Sounds	Ipnos Software	6.1.2 Amazon 6.2 Apple<br><br>Varies with the google device	Health & Fitness	4.7	Free	Yes	5-10 million	2017	10	No	Sleep
myNoise	myNoise BVBA	2.4.2 Apple<br>1.2 google	Health & Fitness	4.5	Free	Yes	50-100k	2017	7	**Mentioned tinnitus**: useful in cases of tinnitus	Noise machine – multiple uses
Tinnitus Therapy Lite	Sound Oasis	1.0.3 Amazon<br>1.1.6 Apple<br>1.1.6 google	Health & Fitness	4.5	Free (only sample of 5 sounds and basic options)	No<br>Pro version?	10-50k	2017	7	**Tinnitus specific:** Tinnitus relief sounds, sound therapy and sound masking to make **tinnitus less noticeable**	Tinnitus

Headspace: Guided Meditation & Mindfulness	Headspace, Inc.	2.2.0 Amazon<br>3.0.3 Apple<br><br>Varies with the google device	Health & Fitness	3.9	Free (only sample of 10 day meditation)	Yes	1-5 million	2017	6	No	Meditation and mindfulness- multiple problems, essentials of living a healthier, happier life
Sleep Bug: White Noise Soundscapes & Music Box	Panzertax	3.4 Apple<br>1.6 Google	Health & Fitness	4.4	Free	Yes	100-500k	2017	6	No	Sleep
Beltone Tinnitus Calmer	Beltone	3.4.2 Apple<br>3.1.4 google	Medical	4.3	Free	No	10-50k	2017	4	**Tinnitus specific**: app uses a combination of sounds and relaxing exercises that aim to **distract your brain from focusing on tinnitus.**	Tinnitus
Sleep Pillow	FITNESS22 LTD	7.4 Apple<br>No version Google	Health & Fitness	4.8	Free	Yes	100-500k	2016	4	No	Sleep
Soothing Sounds Lite	Lost Ego Studios Limited	1.22 Apple<br>1.0 Google	Apple: Medical; Google: Lifestyle	3.5	Free	No	1-5k	2017	4	No	Stress, relaxation
Free Tinnitus	Phase4	1.2 Apple	Medical	NA	Free	No	NA	2016	3	**Tinnitus specific:**	Tinnitus

HQ: Nature sounds to mask ear ringing Changed name to: Tinnitus Aid: help ear ringing	Mobile					Tinnitus HQ pro version of an app available				Recent research shows that tinnitus can be treated by listening to sounds that have been filtered to remove **your specific tinnitus frequencies.***	
Tinnitus Balance	Phonak	2.1 Apple<br>1.1.947 Google	Medical	3.7	Free	No	50-100k	2016	3	**Tinnitus specific:** helps you manage your PTM sound plan.	Tinnitus
Rain Rain Sleep Sounds	Tim Gostony	2.0 Amazon<br>5.2 Apple<br>3.2 Google	Health & Fitness	4.7	Free	Yes	100-500k	2017	3	No	Sleep
Nature Sounds	Relaxio	2.9.3 google	Health & Fitness	4.7	Free	Yes	1-5 million	2016	2	No	Relaxation, sleep
Relax Noise 3	Martin Nathansen	1.6 Amazon<br>1.6 google	Health & Fitness	3.95	Free	No	100-500k	2015	2	**Mentions tinnitus:** masks nerving ambient noise and also **your tinnitus** with white, pink or red background noise.	masking
ReSound Relief	GN ReSound A/S	3.1.5 Apple<br>3.1.4 google	Medical	4	Free	No	10-50k	2017	2	**Tinnitus specific:** app uses a combination of sounds and relaxing exercises that aim to **distract your brain from focusing on tinnitus.** Over	Tinnitus

										time the brain learns to focus less on the tinnitus.	
Sleep Well Hypnosis	Surf City Apps LLC	2.12 Amazon<br>3.9 Apple<br>2.14.4 Google	Health & Fitness	3.8	Free	Yes	100-500k	2017	2	No	Sleep
Zenways	Wimbledon Sound	1.1 Apple	Lifestyle	NA	Free	No	NA	2013	2	No	Relaxation, meditation
Baby Sleep Sounds White Noise	Tiramisu	2.1 Apple<br>2.0.4 Google	Utilities /Parenting	4.3	Free	Yes	100-500k	2017	1	No	Sleep
Bell Sounds	Leafgreen	1.0 Apple<br>1.0 Google	Entertainment	4	Free	No	50-100k	2016	1	No	Entertainment
Binaural Beats	Giorgio Calderolla	1.7 Apple	Health & Fitness	5	Free	Yes	NA	2016	1	No	Relaxation, meditation, sleep, concentration
Brainwave Studio Free	RCS LT	1.6.1 Apple	Health & Fitness	NA	Free	No	NA	2015	1	No	Relaxation, stress relief, sleep support, meditation, mind training
Buddhify	Mindfulness Everywhere LTD	2.7.0 Apple<br>1.0.20 Google	Health & Fitness	4.4	£4.99/£1.99	No	50-100k	2017	1	No	Mindfulness, meditation, increase well being
Chillax	Wimbledon Sound	1.7 Apple	Health & Fitness	NA	Free	No	NA	2017	1	No	Relaxation
Decibel Ultra	Patrick Schaefer	3.3 Apple	Utilities	NA	Free	No	NA	2017	1	No	Sound Meter
Deep Relax	Sonotap	1.5 Apple	Health & Fitness	4	Free	Yes	NA	2013	1	No	Relaxation
Deep Sleep and Relax Hypnosis	Mindifi LLC	1.1 Amazon<br>2.0 Apple	Medical	3.9	Free	Yes	100-500k	2015	1	No	Sleep, relaxation

		1.3 google									
dB	Faber Acoustical, LLC	4.4.2 Apple	Utilities	NA	£0.99	No	NA	2016	1	No	Sound meter
Insight Timer	Insight Network Inc	12.2.21 Apple Varies with google device	Health & Fitness	4.8	Free	No	1-5 million	2017	1	No	Meditation: sleep, anxiety, stress, sleep, recovery from addictions, self-love and compassion
iTinnitus	Calabughi Entertainment Games	1.3 Apple	Medical	NA	£5.99	No	NA	2015	1	**Tinnitus specific:** Tinnitus Retraining Therapy – diagnosis and treatment	Tinnitus
Nature Sounds Relax and Sleep	Zodinplex	2.7 Amazon 2.10 Google	Health & Fitness	4.1	Free	Yes	5-10 million	2016	1	No	Relaxation
NatureScapes	Infinite Wave Media, LLC	3.1 Apple	Health & Fitness	NA	Free	Yes	NA	2015	1	No	Relaxation, focus, sleep
Noisli	Noisli	1.1.4 Apple 1.1.2 google	Health & Fitness	4.3	£1.99/£1.49	No	50-100k	2017	1	No	Relaxation, focus, relieve anxiety
Overcome Tinnitus	Divinity Publishing LTD	51 Apple 1.3 Google	Lifestyle	5	£3.99/£2.99	No	500-100	2015	1	**Tinnitus specific:** you are given a number of post hypnotic and direct suggestions to help you to **overcome all problems associated with tinnitus.**	Tinnitus
Pzizz Deep Sleep & power nap	Pzizz	3.9.5 google	Medical	4.4	Free	No	100-500k	2017	1	No	Sleep

Rain Sounds – Sleep & Relax	Relaxio	Varies with google device	Music & Audio	4.8	Free	Yes	1-5 million	2016	1	No	Relaxation, sleep
Relax Meditation: Sleep Sounds	Ipnos Software	2.5.1 google	Health & Fitness	4.4	Free	Yes	1-5 million	2017	1	No	Relaxation, meditation, sleep
Relax Rain ~ Rain Sounds	mikroid	4.9.3 Amazon<br>Varies with google device	Lifestyle	3.6	£1.99/Free	Yes	1-5 million	2017	1	**Mentions tinnitus:** ideal if you have tinnitus problems (ringing in the ears)	Sleep, meditation concentration, tinnitus
Relaxing Sounds - Improve sleep, relax, meditate	Sonotap	2.6 Apple	Health & Fitness	3.5	Free	No	NA	2017	1	No	Sleep, relaxation, meditation
Sleep Mask - White Noise for Sleep, Relaxation, Concentration	Ultabit, LLC	1.2.1 Apple	Health & Fitness	NA	Free	Yes	NA	2015	1	No	Sleep, relaxation, concentration
SleepStream Classic Lite	Explosive Apps	1.3 Apple	Lifestyle	2.5	Free	No	NA	2011	1	No	Sleep, relaxation, meditation, stress, concentration, enjoyment, noise masking
Smiling Mind	SMILING MIND PTY. LTD.	3.2.1 Apple<br>3.2.1 google	Health and Fitness	3.7	Free	No	100-500k	2017	1	No	Meditation – help bring balance to people's life
Sound Relief	Restored hearing Ltd	1.2.1 Apple	Medical	NA	???	???	NA	2016	1	**Tinnitus specific**: provides tinnitus sound therapy and has been	Tinnitus

										scientifically proven to **reduce tinnitus severity** within the first month of treatment.	
Soundly Sleeping	Three Green Wedges	1.3 Apple<br>1.3 Google	Health & Fitness	3.7	Free	No	500-1000	2016	1	No	Sleep
Starkey Relax	Starkey Hearing Technologies	1.2 Apple<br>1.1.1 Google	Health & Fitness	4.3	Free	No	10-50k	2016	1	**Tinnitus specific**: is an informational resource and self-management tool, which is intended for incorporation into a hearing professional's sound therapy protocol for patients diagnosed with tinnitus**. Sound therapy uses sound to decrease the loudness or prominence of tinnitus.**	Tinnitus
Tinnitus Measurer	Neonix	1.1 Apple	Medical	NA	Free	Yes	NA	2012	1	**Tinnitus specific**: This program designed for measuring tinnitus tone frequency by	Tinnitus

										comparing hearing noise with test signals. **It is useful in measuring and monitoring tinnitus** changes in case the noise has a tone form.	
Tinnitus Therapy Tunes	Dr. Marten	8.30 Google	Medical	4.5	Free	Yes	1-5k	2017	1	**Tinnitus specific**: Current medical research identified regular listening to tailor-made notched music as a novel and **promising therapy for tinnitus** patients. Allows everybody suffering from tinnitus to create their own notched music.	Tinnitus
Tinnitus Trainer	Essential Software Design	1.1 Google	Health & Fitness	3.0	£3.49	No	10-50	2017	1	**Tinnitus specific**: aims to try and teach your brain the difference between the 'false' Tinnitus sounds and real	Tinnitus

										sounds of the same frequency. **Ultimately the aim is to stop Tinnitus from occurring altogether**. Note that results may vary depending on the underlying cause of your Tinnitus.	
White Noise Ambience Lite	logicworks	2.70 Apple	Health & Fitness	NA	Free (Pro version available)	Yes	NA	2017	1	No	Sleep
White Noise Baby	TMSOFT	5.1 Amazon<br>5.1 Apple<br>Varies with google device	Lifestyle	4.5	£0.99/Free	Yes	1-5 million	2016	1	No	Relaxation, sleep, calming babies
White Noise box	Skunk Brothers GmbH	2.4 Apple	Health & Fitness	4	Free	Yes	NA	2014	1	**Mentions tinnitus:** Features quality noise samples helping mask your tinnitus	tinnitus, sleep, concentration, focus, stress
White Noise Generator	Relaxio	1.2.0 Google	Music & Audio	4.8	Free	Yes	500k-1 million	2017	1	No	Relaxation, sleep, concentration
White Noise Market	TMSOFT	6.0.3 Amazon<br>6.0.3 Apple<br>6.0 google	Utilities/Tools	4.5	Free	No	100-500k	2017	1	No	Relaxation, sleep
Widex Zen, Tinnitus Management	Widex A/S	1.0.2 Apple<br>1.0.2 google	Health & Fitness	3.8	Free	No	100-500	2017	1	**Tinnitus specific:** Zen sound therapy	Tinnitus
