# Supplementary material for: Mobile Apps for Management of Tinnitus: Users’ Survey, Quality Assessment, and Content Analysis
Source: JMIR Mhealth Uhealth. 2019 Jan 23;7(1):e10353. doi: 10.2196/10353 (PMC6364200; doi:10.2196/10353)
Supplement: Multimedia Appendix 4 [file mhealth_v7i1e10353_app4.pdf]

Multimedia Appendix 4. Content analysis and features of the 18 apps that at least two respondents listed as those they have tried to manage their tinnitus: (1) White Noise Free, (2) Oticon Tinnitus Sound, (3) Relax Melodies: Sleep Sounds, (4) myNoise, (5) Tinnitus Therapy Lite, (6) Headspace: Guided Meditation & Mindfulness, (7) Sleep Bug: White Noise Soundscapes & Music Box, (8) Beltone Tinnitus Calmer, (9) Sleep Pillow, (10) Soothing Sounds Lite, (11) Tinnitus Aid: Nature sounds to mask ear ringing, (12) Tinnitus Balance, (13) Rain Rain Sleep Sounds, (14) Nature Sounds, (15) Relax Noise 3, (16) ReSound Relief, (17) Sleep Well Hypnosis, (18) Zenways. ***V*** – content/feature present in an app.

**Content/feature**	**1**	**2**	**3**	**4**	**5**	**6**	**7**	**8**	**9**	**10**	**11**	**12**	**13**	**14**	**15**	**16**	**17**	**18**
**Sound generation/sound therapy**	***V***	***V***	***V***	***V***	***V***		***V***	***V***	***V***	***V***	***V***	***V***	***V***	***V***	***V***	***V***		***V***
• Record and loop your own sounds	***V***																	
• Import/download additional sound for free	***V***	***V***		***V***				***V***				***V***				***V***		
• Purchase additional sounds									***V***									
• Direct link to additional sound library app	***V***																	
• Volume control for the sounds	***V***	***V***	***V***	***V***	***V***		***V***	***V***	***V***	***V***	***V***	***V***	***V***	***V***	***V***	***V***		***V***
• Adjusting sound balance	***V***							***V***								***V***		
• Adjusting sound pitch	***V***																	
• Frequency shaping				***V***							***V***							
• Adjusting sound variance	***V***																	
• Adjusting sound intensity (e.g. a small log fire to a roaring beach fire)										***V***								
• Adjusting sound speed	***V***																	
• Frequency shaping options - animating sounds (i.e. zen, subtle, moderate, allegro, wobbler)				***V***														
• Frequency shaping options - setting colour of the sound (brown, grey, pink, white)				***V***														
• Frequency shaping options - setting frequency bandwidth (e.g. centred around specific frequency)				***V***														
• Frequency shaping options - scene (e.g.				***V***														

dark rain, fairy rain, under the leaves for Rain Noise)																		
• Endless sounds	V	V	V	V	V		V	V	V	V		V	V	V	V	V		V
• Looping sounds	V		V									V						
• Advanced soundscape generator which does not loop sounds but generates them in a way that one would not hear the same 10 seconds of sound										V								
• Loop correction (different modes in case the pause could be heard in the looped sounds)			V															
• ‘Long high-quality recordings’											V							
• Mix different sounds to create personalised ‘soundscapes’	V		V				V	V	V	V	V		V			V		
• Adjusting the volume of the mixed sounds individually	V		V				V	V	V	V	V		V			V		
• Adjusting the balance of sound in the mix individually	V							V								V		
• Adjusting the pitch of the sounds in the mix individually	V																	
• Adding random sound effects to the main sound							V							V				
• Rating or marking the favourite sounds and storing them in the favourite folder	V	V						V				V				V		
• User can create personalised sound plan and organise the sounds according to sound type or situations		V						V				V				V		
• Binaural bits or isochronic tones			V	V						V								V
• Play sound in the background while using other apps		V	V	V			V	V	V		V	V	V		V	V		V
• Sound in the context of specific management programme – Progressive												V						

Tinnitus Management																		
**Meditation and mindfulness**			V			V		V								V		V
• Guided meditation			V			V		V								V		V
• Imagery								V								V		
• Guided meditation programmes and single sessions to aid sleep			V															
• Guided meditation sessions to practice techniques for managing stress and tension caused by tinnitus								V								V		
• Wide selection of themed meditations on variety of topics (e.g. depression, self-esteem, stress, cancer, sleep, pregnancy, anxiety)						V												
• Mindfulness of the breath meditation to ‘help relax and find your Zen’																		V
**Relaxation exercises**		V						V								V		
• Breathing exercises		V						V								V		
• Setting the tempo of breathing exercises (deep, slow, normal)								V								V		
• Setting the tempo of breathing exercises (number of breaths per minute)		V																
• Muscle relaxation		V																
**Elements of cognitive behaviour therapy (CBT)**								V								V		
• Changing unpleasant thoughts about tinnitus into something less upsetting								V								V		
• Pleasant activities								V								V		
**Information and education**																		
• Information about tinnitus								V				V				V		
• Using sound for management of tinnitus		V			V			V				V				V		
• Sleep hygiene								V								V		
• What is insomnia and possible causes of																	V	

insomnia																		
• Role of binaural beats				V														
• Role of different frequencies of binaural beats			V															
• What is mediation and mind training						V												
• Weblinks to more information or app help and troubleshooting			V	V	V			V							V	V	V	
• Help Section or brief introduction to an app	V	V			V			V								V	V	
**Hypnosis**																	V	
• Loop sessions																	V	
**Non-auditory stimuli**								V								V		
• Secondary stimuli – colours (choice of 'colour mood')								V								V		
• High quality graphics (as per app description)							V		V		V			V				
**Technical features**																		
• Content can be downloaded and works offline (no streaming required)	V	V	V	V	V	V	V	V	V	V	V	V	V	V	V	V	V	V
• Remote controls to adjust volume while screen is on lock	V	V	V				V	V								V	V	
• Remote controls to pause/start/close the apps while on the screen lock		V	V				V	V								V	V	
• Sharing	V		V								V						V	V
• App community	V			V														
• Advert free		V		V	V	V	V	V				V	V	V	V	V	V	V
• Progress/usage tracking			V			V		V				V				V		
• Available in multiple language options		V	V					V	V	V	V	V	V	V		V		
• Timer for controlling length of sounds or sessions	V	V	V	V	V		V	V	V		V	V	V	V		V	V	V
• Fading out audio option	V										V				V			

• Clock	V		V				V											
• Alarm	V		V							V								
• Date display							V											
• Bedtime reminders			V										V					
